# Development and Characterization of Eudragit^®^-Based Electrospun Nanofibrous Mats and Their Formulation into Nanofiber Tablets for the Modified Release of Furosemide

**DOI:** 10.3390/pharmaceutics11090480

**Published:** 2019-09-17

**Authors:** Marilena Vlachou, Stefanos Kikionis, Angeliki Siamidi, Sotiria Kyriakou, Andrew Tsotinis, Efstathia Ioannou, Vassilios Roussis

**Affiliations:** 1 Section of Pharmaceutical Technology, Department of Pharmacy, School of Health Sciences, National and Kapodistrian University of Athens, 15784 Athens, Greece; asiamidi@pharm.uoa.gr (A.S.); sotiria.kir@gmail.com (S.K.); 2 Section of Pharmacognosy and Chemistry of Natural Products, Department of Pharmacy, School of Health Sciences, National and Kapodistrian University of Athens, 15771 Athens, Greece; skikionis@pharm.uoa.gr (S.K.); eioannou@pharm.uoa.gr (E.I.); 3 Section of Pharmaceutical Chemistry, Department of Pharmacy, School of Health Sciences, National and Kapodistrian University of Athens, 15784 Athens, Greece; tsotinis@pharm.uoa.gr

**Keywords:** furosemide, Eudragit^®^, electrospinning, nanofibers, tablets, modified release

## Abstract

Furosemide, a chloride channel blocker ordinarily used as a high-ceiling or loop diuretic, is practically insoluble in water and dilute acids. Due to its acidic nature, furosemide is mostly absorbed in the stomach and in the upper small intestine. Efforts have focused on the development of sustained release systems of furosemide in order to improve the effectiveness of the drug, which exhibits poor aqueous solubility and poor permeability. Recently, electrospun nanofibrous drug delivery systems have emerged as promising alternative solid-dosage forms due to their advantages of high porosity, high surface to volume ratio, and high drug-loading efficacy. Herein, a number of nanofibrous mats composed of different types of Eudragit^®^ polymers in various concentrations and combinations loaded with furosemide were designed, successfully electrospun, and characterized using SEM, FTIR, DSC, and TGA analyses. The nanofibrous nonwovens were formulated in nanofiber tablets and the release profile of furosemide from them was evaluated at pH 1.2 and 6.8 and compared to that of physical mixture matrix tablets of analogous composition as well as to that of a commercial formulation. It was found that the release of furosemide was compatible with the gastroretentive and slower intestinal release requirements with a well-defined absorption window, while some nanofiber formulations could act as furosemide carriers in emergency situations where a relatively fast onset of its action is required, as in the case of critically ill post-traumatic patients.

## 1. Introduction

**Figure 1:**
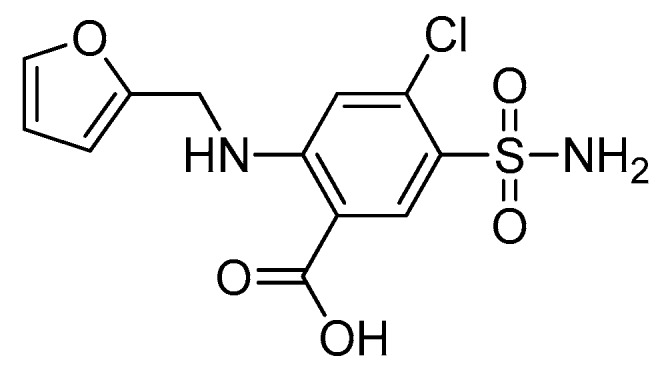
Chemical structure of furosemide.

Furosemide (4-chloro-*N*-furfuryl-5-sulfamoylanthranilic acid, Figure 1), belonging to the chemical class of aminobenzenesulfonamides, is a chloride channel blocker, ordinarily used as a high-ceiling or loop diuretic [1,2,3]. Being a potent loop diuretic, it is used in the treatment of edema related with congestive heart failure, hepatic cirrhosis, and renal disease including nephrotic syndrome as well as to manage hypertension [4]. Its exact mechanism of action is not fully understood [1,4]; however, it has been suggested to inhibit the water reabsorption in the nephron because it blocks the sodium–potassium–chloride co-transporter in the thick ascending limb, which is responsible for the reabsorption. Furthermore, furosemide causes the excretion of calcium, magnesium, and bicarbonate ions [1,4].

Furosemide is practically insoluble in water and dilute acids, while its solubility increases at neutral pH because it is a weakly acidic drug with a pKa of 3.9 [1,2,4,5]. Due to its acidic nature, furosemide is mostly absorbed in the stomach (pH = 1.2) and in the upper small intestine (pH = 6.8–7.4) [1,5]. It is administered per os or parentally with a recommended daily dose of 20–80 mg for adults [6]. Moreover, its half-life in healthy subjects is in the range of 30 to 120 min, and the oral bioavailability varies between 60% and 70% [4,6].

As an active substance with poor aqueous solubility and poor permeability, furosemide belongs to class IV of the Biopharmaceutics Classification System (BCS) [1,2,7]. During the last few years, researchers have tried to develop sustained release systems of furosemide in order to improve the effectiveness of the drug. The efficiency of immediate release versus modified release systems of furosemide in the decrease of fluid (edema) in the thoracic cavity has been studied [8]. Carbopol and sodium alginate have been used, indicating that carbopol can extend the release of furosemide [9]. Different viscosity grades of hydroxypropylmethyl cellulose (HPMC), microcrystalline cellulose, and polyethylene glycol have also been used as excipients [10]. Moreover, the preparation and characterization of solid dispersions of furosemide with Eudragit^®^ L100-55, Eudragit^®^ RL, and RS, achieving variable controlled dissolution profiles according to the different carrier concentrations, have been described [11,12]. In order to provide more effective therapy, devoid of high peak natriuretic and diuretic adverse effects, we recently reported on the modified release profile of furosemide from matrix and compression coated tablets [13].

Electrospun nanofibrous drug delivery systems have lately emerged as promising alternative solid-dosage forms due to their advantages of high porosity, high surface to volume ratio, and high drug-loading efficacy. Electrospinning is a simple, cost-effective, user-friendly, and continuous process of nanofiber production through an electrically charged jet of polymer solution or melt. Among other methods for the fabrication of nanofibers, electrospinning has become the most frequently used method because of its ability to afford nanofibers with unique characteristics such as small pore size, high flexibility in surface functionalities/motifs, and improved mechanical properties. With the appropriate selection of materials (e.g., polymers, solvents, active agent) and electrospinning parameters (e.g., applied voltage, flow rate, tip-to-collector distance), fibers of diverse morphologies such as core-sheath, porous, or hollow structured can be produced [14]. Furthermore, electrospinning is a controlled and reproducible method that has been applied in recent years with increasing frequency for the preparation of controlled-release amorphous solid dispersions and innovative controlled drug delivery systems [15]. Recently, multi-needle or needleless electrospinning techniques have been utilized to increase the range of applications in comparison to conventional electrospinning, while coaxial and tri-axial electrospinning have been used to generate core-shell and multilayer nanofibrous structures with sophisticated structural features [16,17]. Eudragit^®^ polymers have been used in numerous studies for the preparation of nanofibrous systems, whereas advanced materials such as double-pulsatile release core-shell fibers and theranostic fibers for simultaneous imaging and drug delivery have been fabricated [18,19].

In the context of our ongoing interest in the development of more effective controlled release systems for sparingly water soluble drugs [13,20], we designed and characterized nanofibrous electrospun mats composed of different types of Eudragit^®^ polymers in various concentrations and combinations thereof loaded with furosemide. Subsequently, the release profile of furosemide from tablets based on the nanofibrous nonwovens was evaluated and compared to the release profile of furosemide from the respective matrix tablets as well as to that of a commercially available formulation. While the number of reports on the application of nanofibers as amorphous solid dispersions of poorly water-soluble drugs is steadily increasing, studies on the release profile of furosemide incorporated in electrospun nanofibrous systems have not, to the best of our knowledge, been reported so far.

## 2. Materials and Methods

### 2.1. Materials

Furosemide (MW: 330.745 g/mol, 99.6% purity) was purchased from Sigma-Aldrich (Steinheim, Germany). Different grades of Eudragit^®^ polymers (poly(methacrylic acid-*co*-ethyl acrylate) 1:1 (L100-55); methacrylic acid and methyl methacrylate copolymer 1:1 (L100); methacrylic acid and methyl methacrylate copolymer 1:2 (S100); and poly(butyl methacrylate-*co*-(2-demethylaminoethyl) methacrylate-*co*-methyl methacrylate) 1:2:1 (E100)) were purchased from Rohm GmbH Pharma Polymers (Darmstadt, Germany), whereas magnesium stearate was obtained from Riedel-De Haen (Hannover, Germany). Lasix^®^ 40 mg tablets were purchased from a local drug store. All chemicals were of reagent grade and used in the study without further purification.

### 2.2. Electrospinning

Electrospinning was conducted using a γ-High Voltage Research DC power supply generator (Gamma High Voltage Research, Ormond Beach, FL, USA) with a maximum voltage of 50 kV. The polymer solutions were loaded into 10-mL disposable syringes fitted with 23G tip-ground-to-flat needles that were mounted on a horizontally positioned Harvard PHD 2000 programmable syringe pump (Harvard Apparatus, Holliston, MA, USA). The produced nanofibers were deposited on aluminum foil wrapped on a RC-6000 (NaBond Technologies, Hong Kong, China) rotating drum collector at a rotation speed of 400 rpm. Temperature and relative humidity were 20 ± 2 °C and 60 ± 5%, respectively.

### 2.3. Preparation of the Electrospinning Solutions and Fabrication of Micro-/Nanofibrous Mats

All spinning solutions were prepared at room temperature in a EtOH/DMF (8:2) solvent system under stirring for 24 h to ensure homogeneity. Furosemide was added to each polymer solution at an appropriate concentration to afford fiber mats with a 10% total concentration of furosemide. Specifically, the L100, S100, and L100-55 spinning solutions were prepared by dissolving the respective Eudragit^®^ polymers at a concentration of 20% *w*/*v*, followed by the addition of furosemide at 2.2% *w*/*v*. The E100 spinning solution was prepared by dissolving Eudragit^®^ E100 at a concentration of 40% *w*/*v*, followed by the addition of furosemide at 4.4% *w*/*v*. The S100/L100/L100-55 spinning solution was prepared by dissolving S100, L100, and L100-55 at a concentration of 6.7% *w/v* each, followed by the addition of furosemide at a concentration of 2.2% *w*/*v*.

For the fabrication of 1n, 2n, 3n, 4n, and 8n fiber mats (Table 1), the L100, S100, L100-55, E100 and S100/L100/L100-55, spinning solutions were separately electrospun with the applied voltage, tip-to-collector distance, and feeding rate fixed at 25 kV, 15 cm, and 0.5 mL/h, respectively.

For the fabrication of 5n, 6n, 7n, 9n, 10n, and 11n fiber mats (Table 1), the L100, S100 and L100-55 spinning solutions were co-electrospun with the E100 spinning solution on an antiparallel setup with the syringes mounted on two horizontally opposed programmable syringe pumps to ensure a homogeneous blending of the polymer fibers. Applied voltage and tip-to-collector distance were fixed at 25 kV and 15 cm, respectively. For the fabrication of the 5n, 6n, and 7n fiber mats (1:2 *w*/*w* polymer ratio of L100, S100, or L100-55 to E100), the feeding rates of L100, S100, and L100-55 spinning solutions were fixed at 0.5 mL/h, whereas the feeding rate of the E100 spinning solution was adjusted at 0.5 mL/h. In contrast, for the fabrication of the 9n, 10n, and 11n fiber mats (2:1 *w*/*w* polymer ratio of L100, S100, or L100-55 to E100), the feeding rates of the L100, S100, and L100-55 spinning solutions were fixed at 0.5 mL/h, whereas the feeding rate of the E100 spinning solution was adjusted at 0.125 mL/h.

### 2.4. Characterization of Micro-/Nanofibrous Mats

A PhenomWorld desktop scanning electron microscope (SEM, Thermo Fischer Scientific, Waltham, MA, USA) with a tungsten filament (10 kV) and charge reduction sample holder was used for SEM analyses of the nanofibers. The diameters of 100 fibers from each SEM image were measured using the embedded image analysis software (Phenom Pro Suite/Fibermetric, Waltham, MA, USA) and the average fiber diameter was determined. The obtained fiber mats were extracted with ethyl acetate and the recovered residues were analyzed by ^1^H NMR spectroscopy on a Bruker DRX 400 MHz (Billerica, MA, USA) and UV/VIS spectrophotometry on a PerkinElmer Lambda 40 UV/VIS (Waltham, MA, USA) instrument to evaluate the chemical integrity of furosemide after electrospinning. The chemical composition of the fibers was analyzed by Fourier transform infrared spectroscopy (FTIR) using the attenuated total reflection method on a FTIR Bruker Alpha II (Billerica, MA, USA). Differential scanning calorimetry (DSC) analysis was carried out using a TA Thermal Analyzer (Discovery DSC 25, TA instruments, New Castle, DE, USA). Sealed samples of 5–7 mg in aluminum pans were heated at a constant rate of 20 °C/min from 40 to 300 °C under a 25 mL/min flow of nitrogen. Thermogravimetric analysis was performed using a TA Thermogravimetric Analyzer (TGA 55, TA instruments, New Castle, DE, USA) under nitrogen flow of 25 mL/min at a heating rate of 10 °C/min from 40 to 600 °C.

### 2.5. Preparation of Nanofiber Tablets

The nanofiber tablets (Table 1) were produced using a 10 mm diameter die and a hydraulic press (Maassen type, MP 150). The thickness of the tablets was determined on three samples for each batch using a Vernier caliper. Pressure used was 3.5–4.0 tons.

### 2.6. Preparation of Matrix Tablets

The physical mixture matrix tablets (Table 2) were produced using a 10 mm diameter die and a hydraulic press (Maassen type, MP 150). The thickness and hardness tests of the tablets were determined on 10 samples for each batch using a Vernier caliper and a hardness tester (Erweka, type TBH28), respectively. Pressure used was 3.5–4.0 tons.

### 2.7. Dissolution Studies

The in vitro dissolution tests of the nanofiber and physical mixture matrix tablets were performed using a United States Pharmacopeia XXII dissolution apparatus II (Pharmatest, Hainerp, Germany) (paddle method). The dissolution medium for the first 2 h was a buffer solution (450 mL), pH 1.2 (HCl solution 0.2 M) to simulate the stomach pH, and then a buffer solution (450 mL), pH 9 (K_2_HPO_4_ solution, 0.14 M) was added to obtain the required composition of the next phase, which simulated the enteric pH (pH 6.8, final volume 900 mL). The system was maintained at 37.0 ± 0.5 °C and the paddles were rotated at a rate of 50 rpm. Samples (5 mL) were taken at predetermined time intervals and passed through a 0.45 μm cellulose filter. The volume was refilled with an equal amount of fresh medium. The concentration of furosemide released into the medium was measured using a PerkinElmer UV spectrophotometer (Norwalk, CT, USA) at a wavelength of 274 nm (pH 1.2) and 234 nm (pH 6.8).

In order to compare the dissolution profiles of furosemide from each formulation, graphs of the % drug release vs. time were constructed and the *t*_20%_, *t*_50%_, *t*_90%_ values were estimated. The dissolution efficiency (D.E.) %, a parameter used for the estimation of the dissolution, was calculated according to Equation (1) [21]:

(1) $$D.E.=\frac{\int_{t_{1}}^{t_{2}}y\;dt}{y_{100}\left(t_{2}-t_{1}\right)}\times 100$$

where *y* is the percentage of dissolved product and D.E.% is the area under the dissolution curve between time points *t*_1_ and *t*_2_, expressed as a percentage of the curve at a maximum dissolution, *y*_100_, over the same time period.

The in vitro release data were fitted to the Korsmeyer-Peppas equation [22,23]:

(2) $$\frac{M_{t}}{M_{\infty }}=k\times t^{n}$$

where $\frac{M_{t}}{M_{\infty }}$ is the percentage of the released drug substance; *k* is the release rate constant; *t* is the release time; and *n* is the diffusion coefficient. This equation is valid for the first 60% of the fractional release. The *n* values represent either Fickian or non-Fickian release kinetics. Particularly, for the case of cylindrical tablets, *n* > 0.45 corresponds to a Fickian diffusion release (Case I diffusion), 0.45 < *n* < 0.89 to an anomalous transport, *n* = 0.89 to zero order (Case II), and *n* > 0.89 to Super Case II release kinetics [24,25].

## 3. Results and Discussion

Nanofibers are accepted as alternative systems for drug delivery, allowing for the adjustment of the release profiles of active substances with diverse structural features. In the framework of this study, furosemide was successfully incorporated through electrospinning into micro/nano non-woven fibers composed of various types of Eudragit^®^. The chemical integrity of furosemide after the electrospinning process under the applied conditions was verified by ^1^H NMR and UV/VIS spectroscopic analyses of the recovered compound following the extraction of the prepared fiber mats.

**Figure 2:**
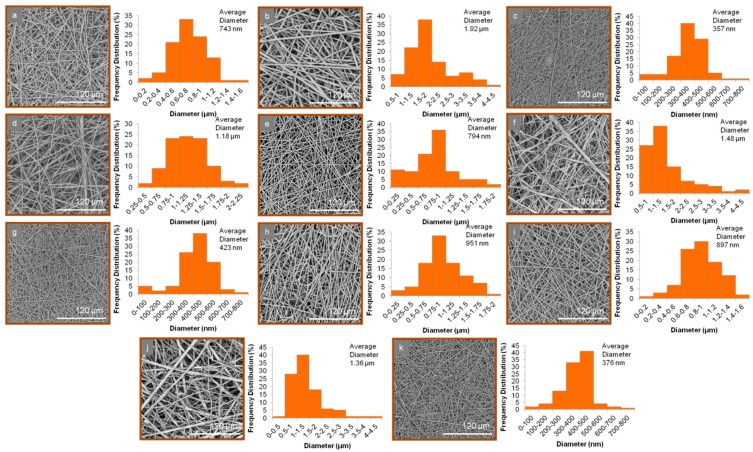
SEM images and average diameter distribution histograms of (**a**) 1n, (**b**) 2n, (**c**) 3n, (**d**) 4n, (**e**) 5n, (**f**) 6n, (**g**) 7n, (**h**) 8n, (**i**) 9n, (**j**) 10n, and (**k**) 11n fibers.

All electrospinning parameters were fine-tuned to obtain uniform, well-shaped fibers. Analysis of the SEM images revealed the morphological characteristics of the produced micro/nanofibrous matrices (Figure 2). In all cases, bead-free fibers of cylindrical shape were observed. The average diameter and size range of the fabricated fibers are shown in Table 3.

**Figure 3:**
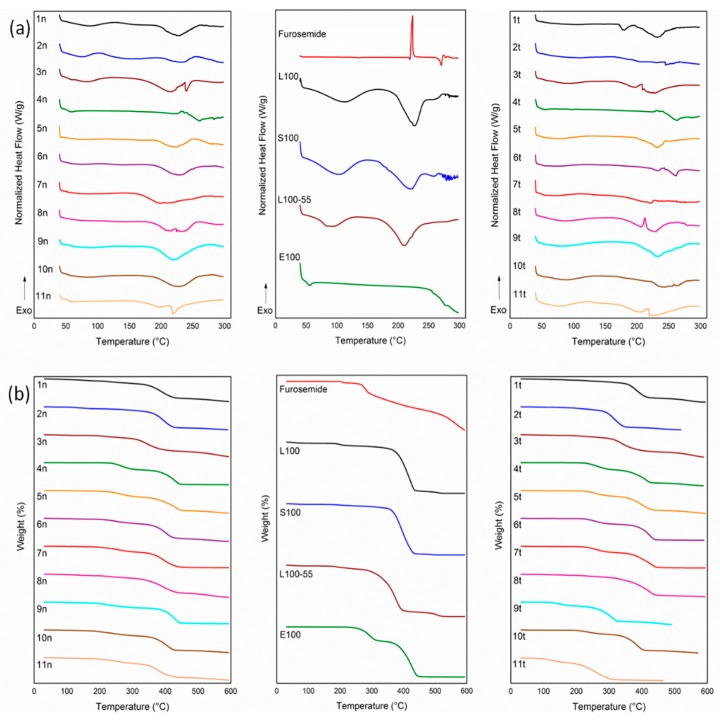
(**a**) DSC and (**b**) TGA thermograms of the raw materials, electrospun nanofibrous mats (formulations 1n–11n) and physical mixture matrix tablets (formulations 1t–11t).

In the DSC analysis (Figure 3a), furosemide, in agreement with the literature [26], showed a weak endotherm at 136.8 °C, a melting endotherm at 219.2 °C, followed by a sharp exothermic peak at 223.7 °C and two endotherms at 270 and 277.5 °C, which were associated with the decomposition phenomena of the drug. The raw Eudragit^®^ polymers, due to their amorphous state, showed broad dehydration endothermic bands below 100 °C, which were followed by broad endotherms over 200 °C due to the decomposition of the polymers. The thermograms of the nanofiber and physical mixture matrix tablets, similar to the raw materials, revealed broad endothermic patterns of dehydration phenomena between 50–100 °C and degradation events over 200 °C, with full decomposition over 300 °C, as also evidenced in their TGA curves (Figure 3b). None of the characteristic thermal events of furosemide were present in the thermograms of the nanofiber and physical mixture matrix tablets, suggesting the molecular interaction of the drug with the polymers and its desired transition into an amorphous state in the designed formulations.

**Figure 4:**
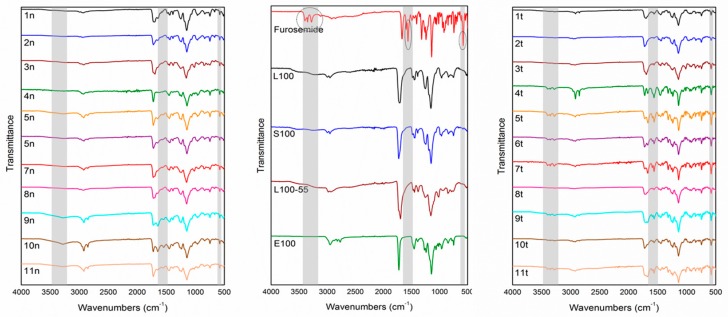
FTIR spectra of the raw materials, electrospun nanofibrous mats (formulations 1n–11n) and physical mixture matrix tablets (formulations 1t–11t).

The FTIR spectrum of furosemide (Figure 4), in agreement with previous studies [26], included two characteristic absorption bands at 3397 and 3282 cm^−1^ ascribed to sulfonamide NH stretching, a secondary amine NH stretching vibration at 3351 cm^−1^, NH bending at 1562–1591 cm^−1^, and characteristic C=O and S=O bands at 1671 and 1141 cm^−1^, respectively. In the FTIR spectra of the L100, S100, L100-55, and E100 polymers, similar characteristic absorption bands were observed. Specifically, the FTIR spectrum of L100 showed a broad –OH absorption band in the range of 3100–3500 cm^−1^, methyl and methylene –CH stretching vibrations at 2997 and 2951 cm^−1^, a characteristic absorption band at 1712 cm^−1^ assigned to –C=O stretching, and –C–O–C stretching vibration at 1154 cm^−1^. Similarly, the FTIR spectrum of S100 revealed a broad –OH absorption band at 3100–3500 cm^−1^, methyl and methylene –CH stretching vibrations at 2997 and 2954 cm^−1^, –C=O stretching at 1727 cm^−1^, and –C–O–C stretching vibration at 1150 cm^−1^, whereas the FTIR spectrum of L100-55 showed broad –OH absorption at 3100–3500 cm^−1^, two absorption bands at 2982 and 2934 cm^−1^ assigned to methyl and methylene –CH stretching vibrations, respectively, –C=O stretching vibration at 1696 cm^−1^, and–C–O–C stretching at 1156 cm^−1^. Slightly different, the FTIR spectrum of E100 revealed methyl and methylene –CH stretching vibration at 2948 cm^−1^, two absorption bands at 2823 and 2769 cm^−1^ ascribed to the dimethylamino group, –C=O stretching vibration at 1725 cm^−1^, and –C–O–C stretching at 1143 cm^−1^.

In the spectra of the designed formulations, the characteristic furosemide bands at 1562–1591 cm^−1^ and at 578 cm^−1^ assigned to aromatic C=C and NH bending and –C–Cl stretching vibrations, respectively, were observed. The characteristic sulfonamido and anilino NH stretching vibrations of furosemide in the 3351–3397 cm^−1^ range, while visible in the FTIR spectra of the physical mixture matrix tablets, were not evident in the spectra of the respective nanofiber formulations. This is possibly due to the fact that both of these functional groups are facilitated to form intermolecular H-bonds only within the nanofibrous matrices, thus leading to considerable broadening of their respective IR bands, making them non-decipherable.

Additionally, small shifts in the IR bands attributed to the –C=O and –C–O–C stretching vibrations of the Eudragit^®^ polymers were observed for the fabricated nanofibers. Specifically, in the FTIR spectra of the L100, S100, L100-55, and E100 polymers, the band due to the –C=O stretching vibration was observed at 1712, 1727, 1696, and 1725 cm^−1^, respectively, whereas in the corresponding nanofibrous matrices, it shifted to 1700, 1721, 1698, and 1721 cm^−1^ for the 1n, 2n, 3n, and 4n formulations, respectively. Similarly, the band attributed to the –C–O–C stretching vibration, observed at 1154, 1150, 1156, and 1143 cm^−1^ in the FTIR spectra of the L100, S100, L100-55. and E100 polymers, respectively, shifted to 1147, 1145, 1157, and 1145 cm^−1^ for the 1n, 2n, 3n, and 4n formulations, respectively. Similar shifts of the corresponding stretching vibrations were observed in the spectra of the remaining nanofibrous matrices, due to the interaction of the polymer matrix with the incorporated furosemide.

**Figure 5:**
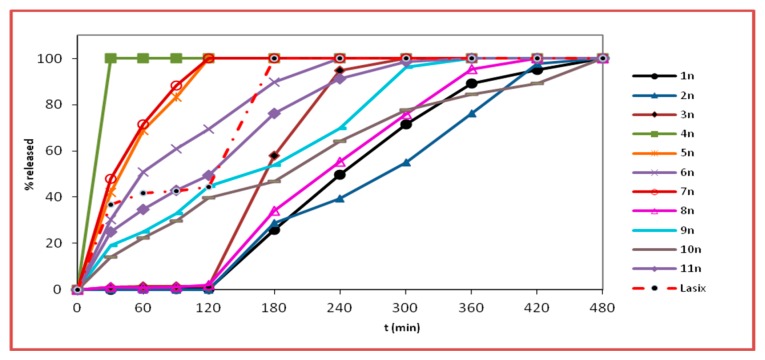
% release (mean values, *n* = 3) (SD < 2) vs. time (min) of furosemide from the nanofiber tablets (formulations 1n–11n) and the commercially available product (Lasix^®^), at pH 1.2 (0–120 min) and at pH 6.8 (120–300 min).

**Figure 6:**
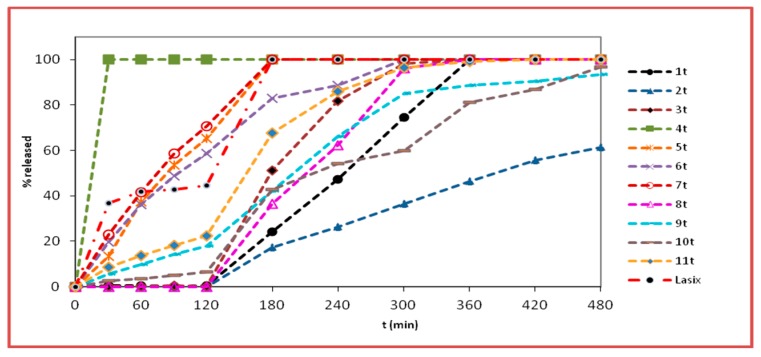
% release (mean values, *n* = 3) (SD < 2) vs. time (min) of furosemide from the physical mixture matrix tablets (formulations 1t–11t) and the commercially available product (Lasix^®^), at pH 1.2 (0–120 min) and at pH 6.8 (120–300 min).

The dissolution curves of furosemide from the nanofiber tablets and physical matrix tablets are depicted in Figure 5; Figure 6, respectively.

The kinetic release properties of the developed formulations 1n–11n and 1t–11t and Lasix^®^ are reported in Table 4. The terms *t*_20%_, *t*_50%_, and *t*_90%_ refer to the time when 20, 50, and 90%, respectively, of the dissolution has been achieved.

**Figure 7:**
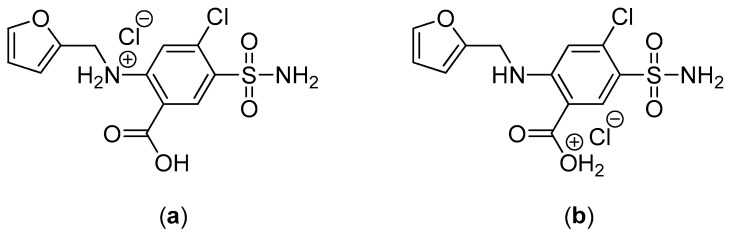
(**a**) Amine-protonated form and (**b**) carboxylic acid-protonated form of furosemide.

In the acidic medium, during the first two hours, no release of furosemide was observed for the nanofiber tablets 1n, 2n, and 3n as well as the physical mixtures matrices 1t, 2t, and 3t. At pH 1.2, the protonation of the anilino-nitrogen of furosemide is preferable to –CO_2_H protonation, even though the proximity of the amine and acid groups seem to allow a simultaneous interaction of the proton with both groups, thus stabilizing and delocalizing the charge more effectively (Figure 7). This would theoretically lead to a non-negligible furosemide solubilization, which, however, was not observed. The absence of release in this case was justifiable by the presence of the Eudragit^®^ polymers used (L100, S100, and L100-55), which are insoluble at pH values lower than 5.5. These results are also corroborated by the fact that furosemide was rapidly released at pH 1.2 from formulations 4n and 4t, which contained the acidic pH-dependent Eudragit^®^ E100. Indeed, at pH 1.2, the release of furosemide from formulations 4n (nanofiber tablets) and 4t (physical mixture matrix tablets) was very fast and completed by *t* = 30 min. It is known that Eudragit^®^ E100 has H-bond acceptor capabilities. This leads to aqueous solubility enhancement of sparingly soluble drugs like furosemide [27]. These findings were also mirrored by the % D.E. values (D.E. = 93.8 for both 4n and 4t).

At pH 1.2, formulations 5n, 6n, and 7n, which contain mixtures of Eudragit^®^ L100, S100, or L100-55 and E100 in a 1:2 ratio, respectively, led to a noteworthy release of furosemide (69.6% to 100% release) when compared with the respective formulations 1n, 2n, and 3n, which did not contain Eudragit^®^ E100. This observation is in agreement with the respective % D.E. values (D.E. of 1n = 51.2, 2n = 43.4, and 3n = 63.2 vs. D.E. of 5n = 89.0, 6n = 81.9, and 7n = 89.6). When the ratio of Eudragit^®^ L100, S100, or L100-55 to E100 was altered to 2:1 (formulations 9n, 10n, and 11n), the release of furosemide was reduced in comparison to formulations 5n, 6n, and 7n. Thus, it seems that the release of furosemide is analogous to the relative percentage of the drug carrier Eudragit^®^ E100 in the mixture of Eudragit^®^ polymers used. Accordingly, the % D.E. values for formulations 9n, 10n, and 11n were 67.1, 59.0, and 74.8, respectively. Furosemide was released slower from the physical mixture matrix tablets when compared to the respective nanofiber tablets. With respect to the relative concentrations of L100, S100, or L100-55 to E100 (1:2 and 2:1), analogous to the nanofiber tablets’ dissolution profiles, were observed (%D.E. of 5t = 81.0, 6t = 76.6, and 7t = 82.4 vs. 9t = 59.9, 10t = 47.9, and 11t = 66.8).

At pH 6.8, the carboxylic acid group of furosemide is converted to COO^−^. Thus, conversely to the pH 1.2 medium (non-ionized COOH), the solubility of the drug is enhanced, and this is mirrored in its release from the physical mixture matrix tablets 1t, 2t, and 3t, and to a greater extent from the nanofiber tablets. Specifically, at pH 6.8, the release of furosemide from the nanofiber tablets 1n, 2n, and 3n reached 100% at 480 min, in the case of the first two, and 100% at 300 min, in the case of 3n. With respect to the release of the drug from the physical mixture matrix formulations 1t, 2t, and 3t, full release (100%) was observed at 360 min from formulations 1t and 3t, while the release of furosemide from formulation 2t was 61.5% at t = 480 min, with the respective %D.E. values of 1n = 51.2, 2n = 43.4, and 3n = 63.2 vs. %D.E. of 1t = 49.6, 2t = 26.6, and 3t = 60.3. Interestingly, furosemide was released slower from formulations 2n and 2t, which both contained Eudragit^®^ S100, than from the formulations containing Eudragit^®^ L100 and L100-55, as has also been previously recorded for the drug mesalazine, which also contains anilino and carboxyl functionalities [28,29]. This could be partly attributed to the much higher viscosity of Eudragit^®^ S100 and possibly due to its capability to form strong H-bonds with furosemide, resulting in the formation of drug-polymer aggregates, which slow down furosemide’s dissolution rate [30]. In addition, the average diameter of the nanofibers in formulation 2n, as observed from the SEM images, seemed to play an important role in the release of furosemide. Indeed, the average diameter of the fibers in formulation 2n was high (1.92 ± 0.46 μm) in comparison to those in the 1n (743 ± 214 nm) and 3n (357 ± 114 nm) formulations, thus resulting in a smaller surface area/volume ratio, leading in turn to a lower release rate [31].

At pH 6.8, the release of furosemide from formulation 6n, which contained a mixture of Eudragit^®^ S100 and Eudragit^®^ E100 in a 1:2 ratio, reached 100% at 240 min, with respect to formulation 2n (100% at t = 480 min), which does not contain any E100 (%D.E. of 2n = 43.4 vs. %D.E. of 6n = 81.9). The release of furosemide from formulations 5n and 7n had already reached 100% in the acidic buffer solution. In all cases when the ratio of Eudragit^®^ L100, S100, or L100-55 to E100 was altered to 2:1 (formulations 9n, 10n, and 11n), the release of furosemide dropped. Therefore, it seems that the presence and the relative percentage of E100 in the Eudragit^®^ mixtures exerts significant impact, modulating the % release of furosemide both at pH 6.8 and 1.2. Analogous results were obtained in the case of the physical mixture matrix tablets (5t, 6t, 7t, 9t, 10t, and 11t), irrespective of the percentage of Eudragit^®^ E100 ratio in the mixtures. However, the release of furosemide was lower in formulations 9t, 10t, and 11t when compared to 5t, 6t, and 7t, respectively.

For formulations 8n and 8t, which contained a mixture of Eudragit^®^ L100, S100, and L100-55, the release of furosemide was not observed in an acidic medium. However, at pH 6.8, the release of furosemide reached 100% from formulation 8n at 420 min and from 8t at 360 min. This marginal difference in the release rate of furosemide from these formulations was also indicated by their %D.E. values (D.E. for 8n = 51.6 and 8t = 55.6). The fact that the release of furosemide from formulation 8n was lower than that observed for formulations 5n and 7n (L100:E100 1:2 and L100-55:E100 1:2, respectively) might be partially affected—aside from the differences in the polymers’ solubilities—by the fact that the average diameter of 8n fibers was relatively higher (951 ± 255 nm) when compared to those of formulations 5n (794 ± 198 nm) and 7n (423 ± 140 nm). The same trend was also observed for the release of furosemide from formulations 9n (L100:E100 2:1, 897 ± 268 nm) and 11n (L100-55:E100 2:1, 376 ± 118 nm) when compared to that of 8n.

In most cases, the nanofiber tablets seemed to follow an anomalous diffusion mechanism as indicated by the *n* value (0.45 < *n* < 0.89) obtained from the Korsmeyer-Peppas equation, indicating that the drug release from these systems might follow both drug diffusion and relaxation of the polymeric matrix (erosion) in an anomalous non-Fickian manner. Most of the physically mixed tablets followed a Super Case release mechanism (see Supplementary Materials). The calculated *n* value corresponding to the release of furosemide from the commercial product Lasix^®^ was 0.11 (*n* < 0.45), indicating that Fickian diffusion was the predominant release mechanism [31].

## 4. Conclusions

In summary, a number of nanofibrous mats composed of different types of Eudragit^®^ polymers in various concentrations and combinations loaded with furosemide were designed, successfully electrospun, and characterized using SEM, FTIR, DSC, and TGA analyses. The nanofibrous nonwovens were formulated in nanofiber tablets and the release profile of furosemide from them was evaluated at pH 1.2 and 6.8 and compared to that of the physical mixture matrix tablets of analogous composition as well as to that of a commercial formulation. It was found that the release of furosemide was compatible with the gastroretentive and slower intestinal release requirements with a well-defined absorption window. Moreover, some nanofiber formulations (e.g., 4n, 5n, and 7n) can act as furosemide carriers in emergency situations [32], where a relatively fast onset of its action is required, as in the cases of critically ill post-traumatic patients [33].

## Figures and Tables

**Table 1:** Composition (in mg) of nanofiber tablets of formulations 1n–11n.

Ingredients	1n	2n	3n	4n	5n	6n	7n	8n	9n	10n	11n
Furosemide	20	20	20	20	20	20	20	20	20	20	20
L100	180				60			60	120		
S100		180				60		60		120	
L100-55			180				60	60			120
E100				180	120	120	120		60	60	60
Total	200	200	200	200	200	200	200	200	200	200	200

**Table 2:** Composition (in mg) of matrix tablets of formulations 1t–11t.

Ingredients	1t	2t	3t	4t	5t	6t	7t	8t	9t	10t	11t
Furosemide	20	20	20	20	20	20	20	20	20	20	20
L100	178				59			59	119		
S100		178				59		59		119	
L100-55			178				59	60			119
E100				178	119	119	119		59	59	59
Mg stearate	2	2	2	2	2	2	2	2	2	2	2
Total	200	200	200	200	200	200	200	200	200	200	200

**Table 3:** Average diameter and size range of the produced fiber mats.

Fiber Mat	Average Diameter	Size Range
1n	743 ± 214 nm	103 nm–1.41 μm
2n	1.92 ± 0.46 μm	597 nm–4.13 μm
3n	357 ± 114 nm	46 nm–738 nm
4n	1.18 ± 0.36 μm	280 nm–2.40 μm
5n	794 ± 198 nm	77 nm–1.83 μm
6n	1.48 ± 0.34 μm	514 nm–4.07 μm
7n	423 ± 140 nm	44 nm–779 nm
8n	951 ± 255 nm	99 nm–1.98 μm
9n	897 ± 268 nm	77 nm–1.83 μm
10n	1.36 ± 0.29 μm	156 nm–4.32 μm
11n	376 ± 118 nm	45 nm–658 nm

**Table 4:** Kinetic release properties of the developed formulations (1n–11n and 1t–11t) and Lasix^®^.

Formulations	*t*_20%_ ± SD ^b^	*t*_50%_ ± SD ^b^	*t*_90%_ ± SD ^b^	D.E.% ^a^ ± SD ^b^
1t	169.7 ± 2.5	245.7 ± 1.2	336.0 ± 2.6	49.6 ± 4.0
2t	199.7 ± 0.9	384.0 ± 1.7	-	26.6 ± 0.1
3t	144.0 ± 0.8	178.7 ± 0.6	269.8 ± 1.3	60.3 ± 3.8
4t	5.7 ± 0.5	15.0 ± 0.6	27.3 ± 1.1	93.8 ± 0.6
5t	39.0 ± 0.8	83.3 ± 1.5	163.3 ± 1.5	81.0 ± 2.3
6t	31.0 ± 1.6	94.7 ± 1.2	247.7 ± 2.1	76.6 ± 2.5
7t	27.3 ± 0.5	75.0 ± 1.0	159.0 ± 1.7	82.4 ± 1.8
8t	153.0 ± 0.8	211.3 ± 0.6	289.0 ± 1.7	55.6 ± 0.6
9t	126.3 ± 1.2	200.3 ± 1.5	404.0 ± 2.6	59.9 ± 2.4
10t	141.7 ± 0.9	219.0 ± 1.7	438.0 ± 1.00	47.9 ± 0.4
11t	104.0 ± 1.4	156.7 ± 0.6	262.7 ± 0.6	66.8 ± 2.9
1n	167.3 ± 1.7	240.3 ± 2.5	368.2 ± 1.8	51.2 ± 3.9
2n	162.3 ± 2.1	281.7 ± 2.1	380.7 ± 1.5	43.4 ± 0.6
3n	140.7 ± 0.5	171.3 ± 2.3	232.0 ± 2.0	63.2 ± 1.4
4n	6.3 ± 1.2	15.0 ± 1.0	27.7 ± 2.5	93.8 ± 0.6
5n	13.3 ± 2.1	39.3 ± 0.6	103.7 ± 1.5	89.0 ± 3.0
6n	21.0 ± 2.4	59.3 ± 1.2	180.7 ± 1.2	81.9 ± 1.4
7n	12.7 ± 0.5	33.3 ± 3.1	95.8 ± 2.8	89.6 ± 1.1
8n	154.0 ± 0.8	224.7 ± 3.5	343.3 ± 1.2	51.6 ± 0.8
9n	36.7 ± 0.9	154.7 ± 1.5	286.3 ± 2.1	67.1 ± 1.7
10n	52.0 ± 0.8	191.7 ± 2.1	424.7 ± 2.5	59.0 ± 0.9
11n	24.7 ± 1.2	121.0 ± 3.6	236.2 ± 1.8	74.8 ± 2.4
Lasix	16.0 ± 0.0	126.0 ± 0.0	169.3 ± 0.6	79.4 ± 0.1

^a^ D.E., dissolution efficiency; ^b^ ± SD, standard deviation.

## Supplementary Materials

The following are available online at https://www.mdpi.com/1999-4923/11/9/480/s1. Table S1: *n* values from Korsmeyer-Peppas equation, Table S2: Plots showing the fits of the from Korsmeyer-Peppas modelling to the data.
