# Supplementary material for: Development and Characterization of Eudragit®-Based Electrospun Nanofibrous Mats and Their Formulation into Nanofiber Tablets for the Modified Release of Furosemide
Source: Pharmaceutics. 2019 Sep 17;11(9):480. doi: 10.3390/pharmaceutics11090480 (PMC6781502; doi:10.3390/pharmaceutics11090480)

pharmaceutics

Supplementary Mateials: Development and Characterization of Eudragit®-based Electrospun Nanofibrous Mats and their Formulation into Nanofiber Tablets for the Modified Release of Furosemide

Marilena Vlachou, Stefanos Kikionis, Angeliki Siamidi, Sotiria Kyriakou, Andrew Tsotinis, Efstathia Ioannou and Vassilios Roussis

Table S1. *n* values from Korsmeyer-Peppas equation.

Formulations	*n* [a]
1t	2.156
2t	1.238
3t	- [b]
4t	- [b]
5t	0.722
6t	0.777
7t	0.798
8t	- [b]
9t	1.497
10t	0.931
11t	2.050
1n	1.897
2n	1.497
3n	- [b]
4n	- [b]
5n	0.615
6n	0.562
7n	0.550
8n	0.624
9n	0.729
10n	0.744
11n	0.693
Lasix	0.131

[a] *n* – diffusion coefficient.

[b] *n* values could not be calculated.

Table S2. Plots showing the fits of the from Korsmeyer-Peppas modelling to the data.

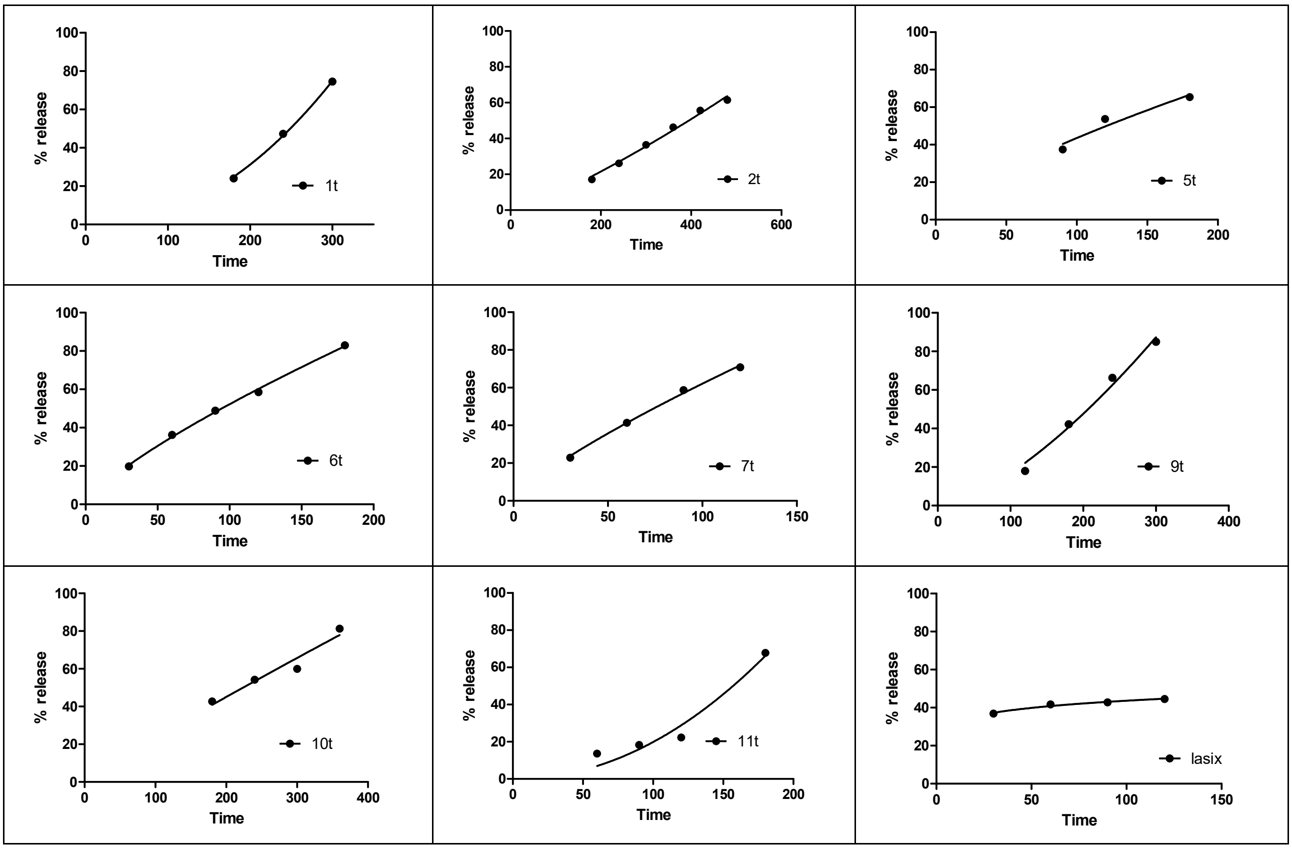

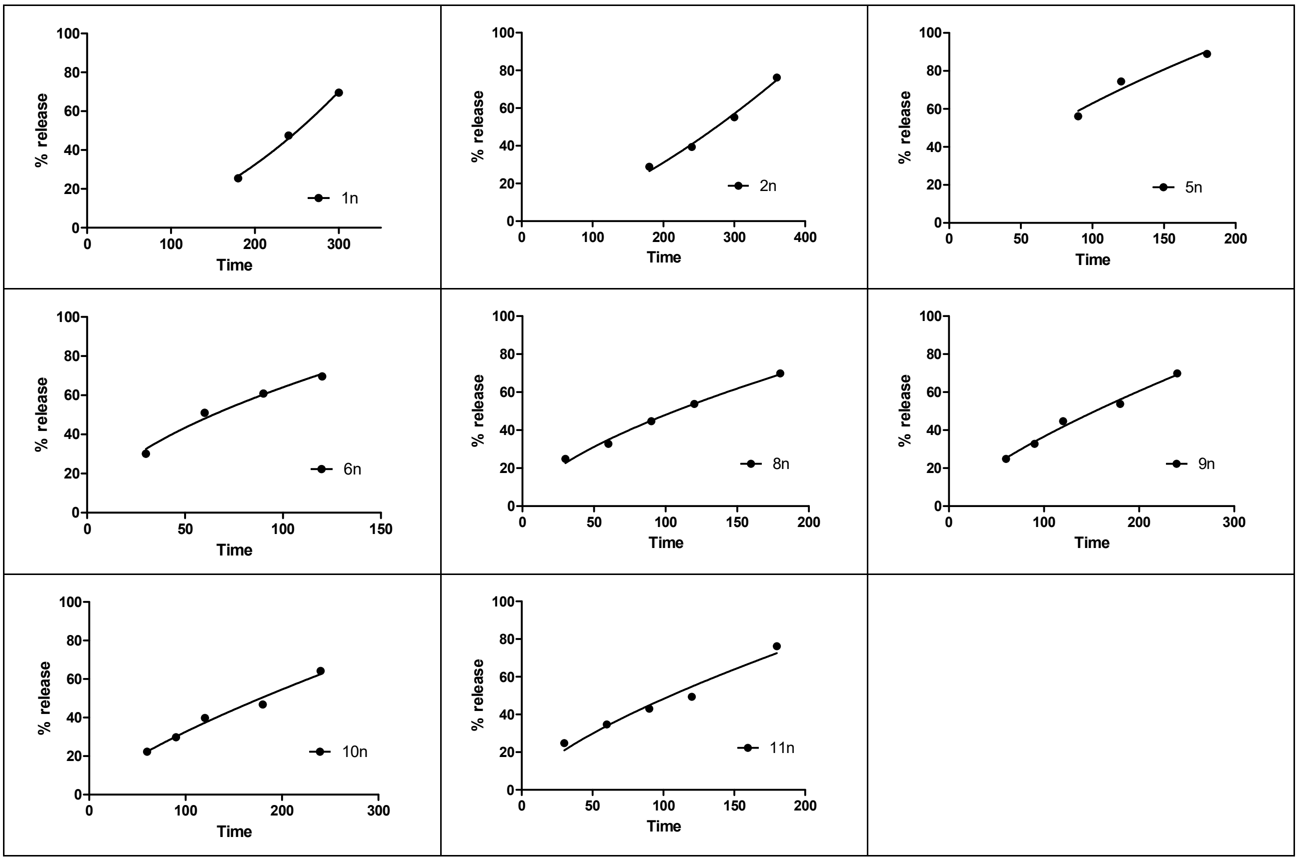

Supplement: Supplementary file 1 [file pharmaceutics-11-00480-s001.pdf]
